# Supplementary material for: The genome sequence of the de Prunner’s Ringlet, Erebia triaria von Prunner, 1798 (Lepidoptera: Nymphalidae)
Source: Wellcome Open Res. 2025 Aug 11;10:425. [Version 1] doi: 10.12688/wellcomeopenres.24693.1 (PMC12423621; doi:10.12688/wellcomeopenres.24693.1)
Supplement: Peer review history

# Reviewer report: Reviewer response for version 1

**DOI:** 10.21956/wellcomeopenres.27209.r129637

**Published:** 2025-09-11

**Authors:** Noah H Rose

**Affiliations:** 1 University of California San Diego, San Diego, California, USA

The present report describes the assembly of a reference genome for Erebia triaria. The authors do a good job providing context for the reference, including alternative names that appear in the literature, noting possibly distinct or vulnerable populations, and highlight the potential for interesting cryptic diversity in the genus. The methods are appropriate, the text is clear, and the assembly appears to be high quality.

Are sufficient details of methods and materials provided to allow replication by others?

Yes

Is the rationale for creating the dataset(s) clearly described?

Yes

Are the datasets clearly presented in a useable and accessible format?

Yes

Are the protocols appropriate and is the work technically sound?

Yes

Reviewer Expertise:

Ecological genomics, evolutionary genetics, vector biology

I confirm that I have read this submission and believe that I have an appropriate level of expertise to confirm that it is of an acceptable scientific standard.

# Reviewer report: Reviewer response for version 1

**DOI:** 10.21956/wellcomeopenres.27209.r129639

**Published:** 2025-09-10

**Authors:** Bin Zhang

**Affiliations:** 1 Qingdao Agricultural University, Qingdao, China

This manuscript presents a high-quality reference genome assembly of *Erebia triaria* (de Prunner’s Ringlet), including chromosome-level assemblies of two haplotypes and the mitochondrial genome. The study is clearly structured, the methods are transparent and replicable, and the raw data and assemblies are openly available. The assembly quality metrics (BUSCO 98.4%, k-mer completeness 99.33%) indicate that the genome is highly reliable and of considerable value for comparative genomics, evolutionary biology, and conservation studies.

 Points for Improvement. 

 1. While quality metrics are well reported, there is limited scientific interpretation. For example, it would strengthen the manuscript to briefly comment on potential chromosome rearrangements, or to compare the assembly with existing Erebia genomes.

 2. Figures such as the Hi-C contact map, Merian element painting, and BUSCO snail plot are useful but under-discussed. A short description of what the plots reveal about genome structure would improve clarity.

 3. Some sentences in the Background section are overly descriptive (e.g., habitat details) and could be streamlined.

Are sufficient details of methods and materials provided to allow replication by others?

Yes

Is the rationale for creating the dataset(s) clearly described?

Yes

Are the datasets clearly presented in a useable and accessible format?

Yes

Are the protocols appropriate and is the work technically sound?

Yes

Reviewer Expertise:

Entomology, Bioinformatics

I confirm that I have read this submission and believe that I have an appropriate level of expertise to confirm that it is of an acceptable scientific standard.

# Reviewer report: Reviewer response for version 1

**DOI:** 10.21956/wellcomeopenres.27209.r129631

**Published:** 2025-08-23

**Authors:** Doga Cedden

**Affiliations:** 1 University of Göttingen, Göttingen, Germany

Chittaro et al. report a high-quality genome assembly from *Erebia triaria. *The background information regarding the lepidopteran is adequate. Sufficient quality control of the genome assembly is provided with standard methodology. The assembly has a BUSCO score of 98.4% and *k*-mer completeness of 99.33% for the combined haplotypes. The raw data and genome are publicly available via the provided accession number. I have no further comments.

Are sufficient details of methods and materials provided to allow replication by others?

Yes

Is the rationale for creating the dataset(s) clearly described?

Yes

Are the datasets clearly presented in a useable and accessible format?

Yes

Are the protocols appropriate and is the work technically sound?

Yes

Reviewer Expertise:

I work on coleopteran pests using RNAi and RNA-seq.

I confirm that I have read this submission and believe that I have an appropriate level of expertise to confirm that it is of an acceptable scientific standard.
